# Integrating Metabolomics and Network Pharmacology to Identify Xanthine Oxidase Inhibitors from Raspberry Leaf: Optimization by Ultrasound-Assisted Extraction and Kinetic Characterization

**DOI:** 10.3390/foods15183231

**Published:** 2026-09-12

**Authors:** Zuoming Cao, Yan Wang, Yonghui Zhang, Zuoting Yang, Jun Sheng, Yang Tian, Lei Peng

**Affiliations:** 1 Key Laboratory of Development and Utilization of Food and Medicinal Resources, Ministry of Education, Yunnan Agricultural University, Kunming 650201, China; 2 Engineering Research Center of Development and Utilization of Food and Drug Homologous Resources, Ministry of Education, Yunnan Agricultural University, Kunming 650201, China; 3 Yunnan Key Laboratory of Precision Nutrition and Personalized Food Manufacturing, Yunnan Agricultural University, Kunming 650201, China; 4 College of Food Science and Technology, Yunnan Agricultural University, Kunming 650201, China

**Keywords:** raspberry leaf, xanthine oxidase, ultrasound-assisted extraction, inhibition kinetics, metabolomics, functional ingredient

## Abstract

The rising global prevalence of hyperuricemia has intensified the search for natural xanthine oxidase (XO) inhibitors from food-grade botanicals. This study screened leaf extracts from four plant species for XO inhibitory activity and identified raspberry leaf as the most potent source. Among seven extraction and pre-treatment methods, ultrasound-assisted extraction (UAE) outperformed conventional decoction and all alternatives. Box–-Behnken response surface methodology optimized UAE parameters to 70 °C, 50 min, and a solid-to-liquid ratio of 1:20 g/mL, yielding an extract with 97.77 ± 0.08% XO inhibition and an IC_50_ of 0.3433 mg/mL—a 5.34-fold potency gain over decoction. Kinetic characterization established a reversible mixed-type inhibition mechanism (*K*_i_ = 0.147 mg/mL), alongside parallel improvements in DPPH, ABTS, and hydroxyl radical scavenging. Widely targeted UHPLC-MS/MS metabolomics identified 2987 metabolites; network pharmacology and molecular docking revealed that the origin of XO inhibition potentially involves terpenoids and coumarins—not the flavonoid and polyphenol fractions conventionally assumed—with dihydroactinidiolide, 7-hydroxy-8-methoxycoumarin, and piperlongumine as the candidate active constituents. These results position UAE-optimized *raspberry* leaf extract as a promising natural functional ingredient for hyperuricemia management and expand the phytochemical scope of plant-derived XO antagonists beyond flavonoids.

## 1. Introduction

Hyperuricemia has emerged as the fourth most prevalent chronic metabolic disorder globally, driven by the interplay of dietary purine overload, impaired renal uric acid excretion, and upregulated endogenous purine catabolism [1,2,3]. At the enzymatic level, xanthine oxidase (XO) catalyzes the terminal two-step oxidation of hypoxanthine to xanthine and subsequently to uric acid, serving as the principal rate-limiting enzyme in purine catabolism [4]. Beyond its canonical role in uric acid generation, XO is a significant source of reactive oxygen species (ROS), including hydrogen peroxide and superoxide anion, which are produced concomitantly during the re-oxidation of its molybdenum cofactor [5,6]. Consequently, XO dysregulation not only elevates circulating uric acid but also intensifies systemic oxidative stress, thereby instigating gout, endothelial dysfunction, renal deterioration, and cardiovascular morbidities [4,5,6]. Targeting XO therefore offers a dual therapeutic benefit: attenuation of uric acid synthesis and concurrent reduction in oxidative burden.

Pharmaceutical XO inhibitors—most notably allopurinol and febuxostat—remain the clinical mainstay for hyperuricemia management. However, chronic administration of these agents is associated with a spectrum of adverse effects, including hepatic and renal injury, severe cutaneous reactions, gastrointestinal intolerance, and, in the case of febuxostat, an elevated cardiovascular mortality risk [7,8]. These safety limitations have prompted interest in alternative XO-suppressive strategies derived from botanical sources, particularly for functional food and dietary supplement applications where long-term, low-dose intake is anticipated [9,10]. It must be emphasized, however, that the “natural equals safe” assumption is a widely held misconception; plant-derived compounds can exhibit hepatotoxicity, renal toxicity, and drug–herb interactions, and their safety profiles require rigorous toxicological evaluation rather than being taken for granted [11,12]. The rationale for exploring botanical XO inhibitors therefore rests not on presumed inherent safety, but on the opportunity to identify food-grade matrices with established dietary histories that may be developed under controlled quality standards.

Among botanical resources, leaf materials generated as agricultural by-products during harvesting and pruning represent an underutilized reservoir of bioactive phytochemicals. Buckwheat (*Fagopyrum esculentum*) leaf, loquat (*Eriobotrya japonica*) leaf, persimmon (*Diospyros kaki*) leaf, and raspberry (*Rubus chingii*) leaf are all consumed in traditional dietary or medicinal contexts and have been reported to contain diverse secondary metabolites, including terpenoids, organic acids, alkaloids, coumarins, flavonoids, and polyphenols [13,14,15,16,17]. Despite this phytochemical richness, the relative XO inhibitory potential of these leaf species has not been systematically compared under standardized extraction and assay conditions. Establishing such a comparative baseline is essential for rationally selecting the most promising feedstock for subsequent process optimization and mechanistic investigation.

Research on plant-derived XO inhibitors has, to date, focused disproportionately on flavonoids and polyphenols, with comparatively little attention directed toward terpenoids, coumarins, and alkaloids [18]. Whether this flavonoid-centric paradigm holds for leaf matrices that are notably rich in non-phenolic constituents remains an open question. *Rubus chingii* leaf, in particular, contains abundant terpenoids and coumarins [14,15], yet rigorous bioactivity profiling, standardized quality control, and refined extraction protocols for this matrix are only beginning to emerge [16,17]. Metabolomics paired with computational target prediction offers a direct, unbiased means to interrogate which compound classes–beyond the traditionally assumed flavonoid and polyphenol fractions–actually contribute to XO inhibition.

Extraction method critically governs the recovery, stability, and compositional profile of bioactive compounds [19]. Conventional water decoction is straightforward but suffers from low extraction efficiency, prolonged operation times, and thermal degradation of heat-sensitive constituents [20,21]. Ultrasound-assisted extraction (UAE) disrupts plant cell walls via acoustic cavitation, enabling enhanced compound release under milder temperature conditions that are compatible with food-processing requirements [22]. Box–Behnken response surface methodology permits rapid multi-variable optimization with minimal experimental runs and has been successfully applied to the extraction of bioactive compounds from plant materials [23,24]. Nevertheless, no study has yet optimized UAE parameters specifically for XO-inhibitory activity from *R. chingii* leaves, nor has the inhibitory mechanism of the optimized extract been elucidated.

We therefore hypothesized that (i) among four leafy species, *R. chingii* leaf would exhibit the strongest XO inhibitory activity; (ii) XO inhibition by the optimized extract derives primarily from terpenoids, coumarins, and alkaloids rather than from the flavonoid and polyphenol fractions conventionally assumed to dominate plant-derived XO antagonism; and (iii) UAE would concentrate these non-phenolic actives more effectively than conventional decoction. To test these hypotheses, we screened buckwheat, loquat, persimmon, and raspberry leaves for XO inhibition under uniform conditions, evaluated the influence of geographical origin and pre-treatment strategy, optimized UAE by single-factor and Box–Behnken experiments, characterized inhibition kinetics, assessed antioxidant capacity, and employed UHPLC-MS/MS widely targeted metabolomics, network pharmacology, and molecular docking to identify active constituents and their binding modes. This work provides mechanistic and technical support for valorizing raspberry leaf—an agricultural by-product—as a source of natural, food-grade XO inhibitors for hyperuricemia management.

## 2. Materials and Methods

### 2.1. Materials and Reagents

Four leaf types underwent screening in this work: buckwheat (*Fagopyrum esculentum*), loquat (*Eriobotrya japonica*), persimmon (*Diospyros kaki*), and raspberry (*Rubus chingii* Hu, Rosaceae). Buckwheat, loquat, and persimmon leaves were purchased as food-grade dried slices from a local specialized market in Kunming, Yunnan Province, China. Raspberry leaves were procured in December 2025 as food-grade dried products via certified e-commerce platforms, sourced from four provinces—Jiangsu, Guangxi, Anhui, and Zhejiang—and authenticated as dried Rubus chingii Hu (Rosaceae) leaves. Voucher specimens were deposited at the College of Food Science and Technology, Yunnan Agricultural University.

All chemical reagents were of analytical or higher grade unless otherwise specified. Xanthine (≥98.0% purity, CAS 69-89-6) and allopurinol (≥99.0% purity, CAS 315-30-0) were obtained from Aladdin Bio-Chem Technology Co., Ltd. (Shanghai, China). Xanthine oxidase (from bovine milk, 10.8 U/mg protein, CAS 9002-17-9) and phosphate-buffered saline (PBS, pH 7.4) were procured from Shanghai Yuanye Bio-Technology Co., Ltd. (Shanghai, China) and Biosharp Life Sciences (Hefei, China), respectively. For the antioxidant assays, 2,2-diphenyl-1-picrylhydrazyl (DPPH, ≥95.0%, CAS 1898-66-4), 2,2′-azino-bis (3-ethylbenzothiazoline-6-sulfonic acid) diammonium salt (ABTS, ≥98.0%, CAS 30931-67-0), potassium persulfate (K_2_S_2_O_8_, ≥99.5%, CAS 7727-21-1), salicylic acid (≥99.5%, CAS 69-72-7), ferrous sulfate heptahydrate (FeSO_4_·7H_2_O, ≥99.0%, CAS 7782-63-0), hydrogen peroxide (H_2_O_2_, 30% *w*/*w* aqueous solution, CAS 7722-84-1), and L-ascorbic acid (Vitamin C, ≥99.0%, CAS 50-81-7) were purchased from SYYBIO Technology Co., Ltd. (Shanghai, China) and Sinopharm Chemical Reagent Co., Ltd. (Shanghai, China), respectively. Ethanol absolute (≥99.7%, CAS 64-17-5) was obtained from Sinopharm Chemical Reagent Co., Ltd. (Shanghai, China). Acetic acid (glacial, ≥99.5%, CAS 64-19-7) and sodium bicarbonate (NaHCO_3_, ≥99.5%, CAS 144-55-8) of food-grade quality were obtained from Sinopharm Chemical Reagent Co., Ltd. (Shanghai, China). Pectinase (from Aspergillus niger, 300 U/mL, food-grade) was supplied by Solarbio Science & Technology Co., Ltd. (Beijing, China).

For chromatographic analysis, methanol (LC-MS grade, CAS 67-56-1), acetonitrile (LC-MS grade, CAS 75-05-8), formic acid (LC-MS grade, ≥98.0%, CAS 64-18-6), and ammonium acetate (LC-MS grade, ≥98.0%, CAS 631-61-8) were furnished by Merck KGaA (Darmstadt, Germany). Internal standards for metabolomics analysis were provided by the MetWare Biotechnology Co., Ltd. (Wuhan, China). All experiments relied on ultrapure water prepared with a Milli-Q Direct 8 water purification system (Millipore, Billerica, MA, USA; resistivity ≥ 18.2 MΩ·cm).

### 2.2. Xanthine Oxidase Inhibitory Activity and Half-Maximal Inhibitory Concentration (Ic_50_) Determination

Inhibition of XO was quantified spectrophotometrically following established protocols [4,8]. Briefly, 50 μL of sample solution was mixed with 50 μL of XO solution (0.05 U/mL, freshly prepared in 50 mmol/L phosphate-buffered saline, pH 7.5) and pre-incubated at 37 ± 0.5 °C for 5 min [4]. The reaction was initiated by adding 150 μL of xanthine solution to achieve a final substrate concentration of 1.0 mmol/L [8]. This concentration is well above the Michaelis constant (K_m_) of XO for xanthine (reported K_m_ ≈ 5–30 μmol/L under comparable assay conditions), ensuring substrate saturation and zero-order kinetics with respect to xanthine. The final reaction volume was 250 μL. After incubation for 30 min at 37 ± 0.5 °C, absorbance was measured at 290 nm, and the XO inhibitory rate was calculated [8].

A series of gradient concentrations was prepared, and the dose–response curve was fitted by four-parameter logistic nonlinear regression using GraphPad Prism 9.5.1 to obtain the IC_50_ value. Allopurinol was assayed in parallel as the positive control under identical conditions.

The XO inhibitory rate was calculated according to Equation (1):

XO inhibition rate (%) = [1 − (D_1_ − D_2_)/(D_3_ − D_4_)] × 100% (1)

where D_1_ is defined as the absorbance of the complete reaction mixture (sample + XO); D_2_ as the absorbance of the sample-containing well lacking enzyme; D_3_ as the absorbance of the reagent blank (buffer + XO); and D_4_ as the absorbance of the blank without enzyme.

### 2.3. Screening of Xanthine Oxidase Inhibitory Active Components

#### 2.3.1. Preparation of Conventional Decoction Extract

Raspberry leaves were pulverized and passed through a 40-mesh sieve (aperture 0.425 mm). The powder was sealed and stored in a desiccator containing silica gel desiccant, protected from light, at room temperature (25 ± 2 °C) until use. An accurately weighed 200.0 g of the powder was placed in a fully automatic herbal medicine decoction machine (model YJW/2+1, Cangzhou Penglin Pharmaceutical Equipment Co., Ltd., Cangzhou, China; rated capacity 20 L). Ultrapure water (2.0 L) was added at a solid-to-liquid ratio of 1:10 (m/V), giving an actual liquid volume of approximately 10% of the equipment rated capacity to ensure sufficient headspace for boiling reflux. The mixture was soaked at ambient temperature for 30 min. Decoction parameters were set at 95 °C, slight pressure 0.1 MPa, for 30 min. After decoction, the extract was filtered through four layers of medical gauze. The filtrate was centrifuged at 4 °C for 15 min at 4000× *g*, and the supernatant was collected as the crude extract [25,26]. The supernatant was concentrated by rotary evaporation under reduced pressure.

To determine the solids content required for calculating extract concentration, a 10.0 mL portion of the concentrate was placed in a tared flat-bottom dish and dried to constant mass in a vacuum oven at 37 °C (vacuum ≤ 100 Pa). Vacuum oven-drying at this temperature was chosen for gravimetric analysis because it is a rapid, established analytical method for moisture determination that provides reproducible constant-mass endpoints suitable for concentration calculations. The remainder of the concentrate was lyophilized to obtain the dried extract used for all subsequent bioactivity assays and metabolomic analysis. Freeze-drying was selected for the bulk material because it minimizes thermal degradation of heat-labile bioactive constituents—particularly terpenoids and coumarins, which were subsequently identified as the principal xanthine oxidase inhibitors (Section 3.5)—that might be compromised by prolonged exposure to even mild heat during vacuum oven-drying. Thus, the two drying methods served distinct purposes: vacuum oven-drying was used solely for analytical gravimetry of a small aliquot, whereas freeze-drying was applied to the bulk sample to preserve bioactive integrity for functional assays.

Concentrations are expressed as mg of dried extract per mL based on the gravimetric analysis of the sample. Solutions were maintained at 4 °C until needed.

#### 2.3.2. Screening of Four Leaf Materials

Each plant material was pulverized, passed through a 40-mesh sieve (aperture 0.425 mm), and processed into crude extracts as described in Section 2.3.1. The extracts were re-dissolved in ultrapure water at 3.0 mg/mL (on a dry extract basis). XO inhibitory activity was measured and IC_50_ values were calculated following the procedure in Section 2.3.1. All determinations were performed in triplicate; the material with the lowest IC_50_ was selected for further study.

#### 2.3.3. Screening of Raspberry Leaves from Different Geographical Origins

*Raspberry* leaves from Jiangsu, Guangxi, Anhui, and Zhejiang provinces were pulverized, sieved, and subjected to crude extract preparation as described in Section 2.3.1. The extracts were reconstituted in ultrapure water at 2.5 mg/mL (on a dry extract basis), and XO inhibitory activity and IC_50_ values were determined. Experiments were conducted in triplicate. The geographical origin yielding the lowest IC_50_ was selected for subsequent extraction optimization.

#### 2.3.4. Screening of Seven Pre-Treatment Methods

To systematically evaluate the effect of pre-treatment strategies on the XO inhibitory activity of raspberry leaf, seven representative methods were compared, encompassing physical disruption (ultrasound-assisted, low-temperature freezing), thermal processing (steaming, high-pressure steaming), chemical modification (acid treatment, alkaline treatment), and enzymatic hydrolysis (pectinase treatment). Raspberry leaf powder from Jiangsu province was subjected to each pre-treatment, followed by extraction as described in Section 2.3.1. The extracts were reconstituted in ultrapure water at 2.0 mg/mL (on a dry extract basis), and XO inhibitory activity and IC_50_ values were determined. All treatment groups were compared against a non-pre-treated conventional decoction control, and the relative inhibitory rate was calculated. Experiments were performed in triplicate.

Steaming: The powder was steamed at 100 °C for 60 min [27].

Ultrasound-assisted extraction: Powder samples were combined with ultrapure water at a solid-to-liquid ratio of 1:10. Sonication was carried out inside an ultrasonic bath under the conditions of 200 W power and 40 kHz frequency, maintaining 70 °C for 30 min [28,29].

Low-temperature freezing treatment: The powder was stored at −20 °C over 24 h, and then allowed to thaw naturally at ambient temperature [30].

Acid treatment: Powder materials were immersed in acetic acid solution adjusted to pH 3.5, and kept at ambient temperature for 60 min [31].

Alkaline treatment: Samples were steeped in sodium bicarbonate solution (pH 8.5) at ambient temperature with a treatment duration of 120 min [32].

Enzymatic treatment: The powder was homogenized with ultrapure water at a solid-to-liquid ratio of 1:10. Pectinase was supplemented to the suspension, with an enzyme activity of 300 U/mL. One unit of pectinase activity corresponds to the liberation of 1 μmol galacturonic acid per hour at 45 °C and pH 4.5. Afterwards, the resulting mixture was incubated within a shaking water bath at 45 °C for 60 min. Enzyme deactivation was subsequently performed by heating the mixture in a boiling water bath for 5 min [33].

High-pressure steaming: The powder was treated in an autoclave at 0.25 MPa and 120 °C for 15 min [34,35].

### 2.4. Ultrasound-Assisted Extraction Optimization

#### 2.4.1. Single-Factor Experiments

Each of three extraction variables—ultrasound temperature (40, 50, 60, 70, 80 °C), ultrasound duration (20, 30, 40, 50, 60 min), and solid-to-liquid ratio (1:5, 1:10, 1:15, 1:20, 1:25, g/mL) —was explored separately. XO inhibition measured at 1.8 mg/mL provided the benchmark for narrowing down a viable operating window for every factor.

#### 2.4.2. Response Surface Methodology Optimization

We built a Box–Behnken design (three factors, three levels) from single-factor results, using XO inhibition rate as the response. Seventeen runs included 5 center points in randomized order. Extracts were assayed at 1.5 mg/mL to avoid saturation. Preparation and analysis followed Section 2.3.1 (Supplementary Table S1).

### 2.5. Xanthine Oxidase Inhibition Type and Kinetic Analysis

Assays tracked inhibition kinetics after published methods [36,37,38] with small changes. XO ranged from 0.025 to 0.15 U/mL, extract from 0.15 to 0.45 mg/mL, and xanthine stayed at 1.0 mmol/L. To test reversibility, we plotted reaction rate against enzyme concentration. Lineweaver–Burk plots (substrate 0.25–1.5 mmol/L) gave the inhibition type, and Dixon plots at 0.5 and 1.0 mmol/L substrate gave *K*_i_. GraphPad Prism 9.5.1 fitted curves and drew plots.

### 2.6. Antioxidant Activity Analysis

Free-radical quenching capacity was probed through three complementary assays—DPPH, ABTS, and hydroxyl radical scavenging—following literature protocols [39,40,41] with adaptation. For the DPPH radical-scavenging test, 50 μL sample solution was mixed with 150 μL ethanolic DPPH· solution (0.3 mmol/L). The resultant mixture was incubated under dark conditions for 30 min, and absorbance of the remaining radicals was determined at 517 nm. Regarding the ABTS assay, ABTS cation radicals were produced by reacting 7 mmol/L ABTS solution with 2.45 mmol/L potassium persulfate solution. After 12 h dark incubation, the prepared stock solution was diluted until its absorbance reached 0.70 ± 0.02 at 734 nm. Subsequently, 50 μL sample was added to 150 μL of the diluted radical working solution. After 6 min of light-avoidant incubation, absorbance readings were captured at 734 nm. The Fenton-reaction-based system was applied to evaluate hydroxyl-radical-eliminating activity. Briefly, samples were mixed with FeSO_4_, ethanol-dissolved salicylic acid and H_2_O_2_ sequentially. The reaction system was kept at 37 °C for 30 min, and the generated chromogenic product was measured spectrophotometrically at 510 nm. L-Ascorbic acid served as the positive control across all three antioxidant measurements.

The percentage scavenging was computed using Equation (2):

Scavenging rate (%) = [1 − (A_1_ − A_2_)/A_0_] × 100% (2)

where A_0_ refers to the absorbance value of reagent blank; A_1_ represents the absorbance recorded for sample reaction system; and A_2_ corresponds to the absorbance of sample blank group. IC_50_ parameters were derived by fitting concentration–inhibition curves.

### 2.7. Widely Targeted Plant Metabolomics Analysis

#### 2.7.1. Sample Preparation

A 50 mg aliquot of the freeze-dried UAE-optimized extract was transferred into a 2 mL centrifuge tube, followed by supplementation with 1000 μL of methanol–acetonitrile–water solution containing internal standards. The resulting mixture was initially subjected to vortex mixing, subsequently homogenized using a ball mill, and finally ultrasonicated for a short period in an ice-water bath to ensure thorough cell disruption. After centrifugation at 12,000× *g* for 15 min at 4 °C, the precipitated insoluble debris was discarded, and the clarified supernatant was filtered through a 0.22 μm PTFE membrane before being loaded onto the LC-MS system. To enable quality-control monitoring, equal volumes of every filtered sample were pooled together to generate a single mixed QC sample.

#### 2.7.2. Chromatography and Mass Spectrometry Conditions

Chromatographic separation was carried out using an ACQUITY UPLC HSS T3 column (100 mm × 2.1 mm, 1.8 μm; Waters, Milford, MA, USA), with column temperature stabilized at 40 °C. The mobile-phase system comprised solvent A (0.1% formic acid in water supplemented with 5 mM ammonium acetate) and solvent B (acetonitrile containing 0.1% formic acid). The same mobile-phase mixtures were applied for both positive-ion and negative-ion acquisition modes. The gradient elution profile was configured as described below: starting with 2% B and sustaining this condition for 1.5 min; linearly raising the proportion of B up to 50% at 5 min and keeping this state for 4 min; further linear ramp-up to 98% B at 9 min (1 min hold); switching back to 2% B within 10.00–11.00 min, followed by re-equilibration until 14 min. The flow velocity was set to 0.35 mL/min, and 4 μL of sample solution was injected for each run.

Metabolite signals were acquired with a Qtrap 6500+ mass spectrometer (AB Sciex, Framingham, MA, USA) fitted with a Turbo ion-spray ESI source. Data collection was managed via Analyst 1.6.3 software. The ion-source temperature was kept constant at 550 °C; electrospray voltages were adjusted to +5500 V for positive-ion mode and −4500 V for negative-ion mode. The values of GS1, GS2 and CUR gas pressures were 50 psi, 55 psi and 35 psi, respectively. Collision-induced dissociation was operated under medium energy level. All measurements were completed under multiple reaction monitoring (MRM) mode. Before sample batch injection, MRM ion transitions were predefined through direct infusion of reference standards whenever accessible. For each ion pair, declustering potential (DP) and collision energy (CE) were individually fine-tuned to achieve optimal signal responses. Throughout data acquisition, target MRM ion pairs were tracked within scheduled time windows matching the elution interval of each individual metabolite.

#### 2.7.3. Metabolite Identification and Quantification

Metabolite identification was performed by matching the acquired MS/MS spectral data against the in-house GB-PLANT metabolite database (MetWare Biotechnology Co., Ltd., Wuhan, China). A metabolite was positively identified when its characteristic precursor ion (Q1), product ion (Q3), and chromatographic retention time (RT) matched the corresponding entries in the database. The identified metabolite list was manually curated to remove redundancies and isotopic adducts.

For quantification, MRM peak areas were extracted using Analyst 1.6.3 software. To correct for inter-sample variation in ionization efficiency and instrument response, peak areas of the same metabolite across different samples were normalized by internal-standard correction and QC-based inter-sample correction following the procedure described by Fraga et al. [42]. Metabolite abundance is reported as relative quantification values based on corrected peak–area ratios.

### 2.8. Network Pharmacology and Molecular Docking

Candidate compound datasets originated from UHPLC-MS/MS metabolomics results. SwissADME webserver (http://www.swissadme.ch (accessed on 7 April 2026)) was adopted to screen these candidates according to predicted oral bioavailability (OB) and drug-likeness (DL) properties. Only chemical entities satisfying the cutoff values of OB ≥ 30% and DL ≥ 0.18 proceeded to subsequent target-forecasting procedures. Potential molecular targets of qualified compounds were forecast via two online platforms: SwissTargetPrediction (http://www.swisstargetprediction.ch (accessed on 7 April 2026), probability > 0.1) and PharmMapper (http://www.lilab-ecust.cn/pharmmapper (accessed on 7 April 2026), fit score > 5.0). Target outputs obtained from both resources were merged and redundant entries were eliminated. Genes closely associated with hyperuricemia were collected from GeneCards (https://www.genecards.org (accessed on 7 April 2026)) and OMIM (https://omim.org (accessed on 7 April 2026)) databases with “hyperuricemia” set as retrieval term. Overlapping entries between compound-related putative targets and disease-associated genes constituted the shared-target dataset.

These intersected targets were submitted to the STRING database (https://string-db.org (accessed on 7 April 2026), version 12.0) to build the protein–protein interaction (PPI) network, with species limited to Homo sapiens and medium-confidence interaction score (0.4) as the filtering threshold. The constructed PPI network was further visualized and processed within Cytoscape software (version 3.9.1). The CytoHubba plugin was utilized to screen hub targets on the basis of degree centrality metric; the top 10 nodes possessing the greatest connectivity values were regarded as hub genes. GO functional annotation alongside KEGG pathway enrichment analyses were completed by Metascape (https://metascape.org (accessed on 7 April 2026)) under default parameter settings, and statistical significance was set at *p* < 0.05.

Regarding molecular docking simulation, the crystal structure of xanthine oxidase (PDB ID: 3NVY, resolution = 2.20 Å) was downloaded from the RCSB Protein Data Bank. Pre-processing of the protein receptor was accomplished in AutoDockTools (ADT, version 1.5.6): residual water molecules and intrinsic co-crystallized ligands were deleted, polar hydrogen atoms were supplemented, and Gasteiger partial charges were allocated. Three-dimensional structures of candidate molecules were generated by Chem3D (version 20.0), followed by energy minimization adopting the MM2 force field (10,000 iteration cycles, RMS gradient convergence threshold: 0.001 kcal/mol·Å). A 60 × 60 × 60-point grid box with 0.375 Å grid spacing was arranged surrounding the molybdopterin catalytic pocket, whose central coordinate referred to the position of native co-crystallized ligand. Docking computation was implemented in AutoDock 4.2 applying the Lamarckian genetic algorithm (LGA). Key algorithm parameters were configured as follows: population size 150, maximal energy evaluation count 2,500,000, maximum generation 27,000, crossover rate 0.8, mutation rate 0.02, elitism value 1, local-search iteration 300. For every test ligand, one hundred separate docking computations were carried out. The conformation presenting the lowest binding free energy (ΔG) was chosen as the optimal binding pose. PyMOL (version 2.5.0) was employed to visualize binding conformations and generate molecular interaction diagrams.

### 2.9. Statistical Analysis

Each determination was carried out with three independent replicates, and data are reported as mean ± standard deviation. Group differences were assessed by one-way ANOVA in SPSS 25.0, with post-hoc separation using Duncan’s multiple range test (*p* < 0.05). Second-order models for the response surface were constructed in Design-Expert 13.0; their adequacy was checked by ANOVA, and goodness-of-fit was judged from the F statistic together with the lack-of-fit test. Dose–response curves were fitted by four-parameter logistic regression in GraphPad Prism 9.5.1 to derive IC_50_ values.

## 3. Results and Discussion

### 3.1. Screening of Raw Materials, Geographical Origins, and Pre-Treatment Methods

**Figure 1:**
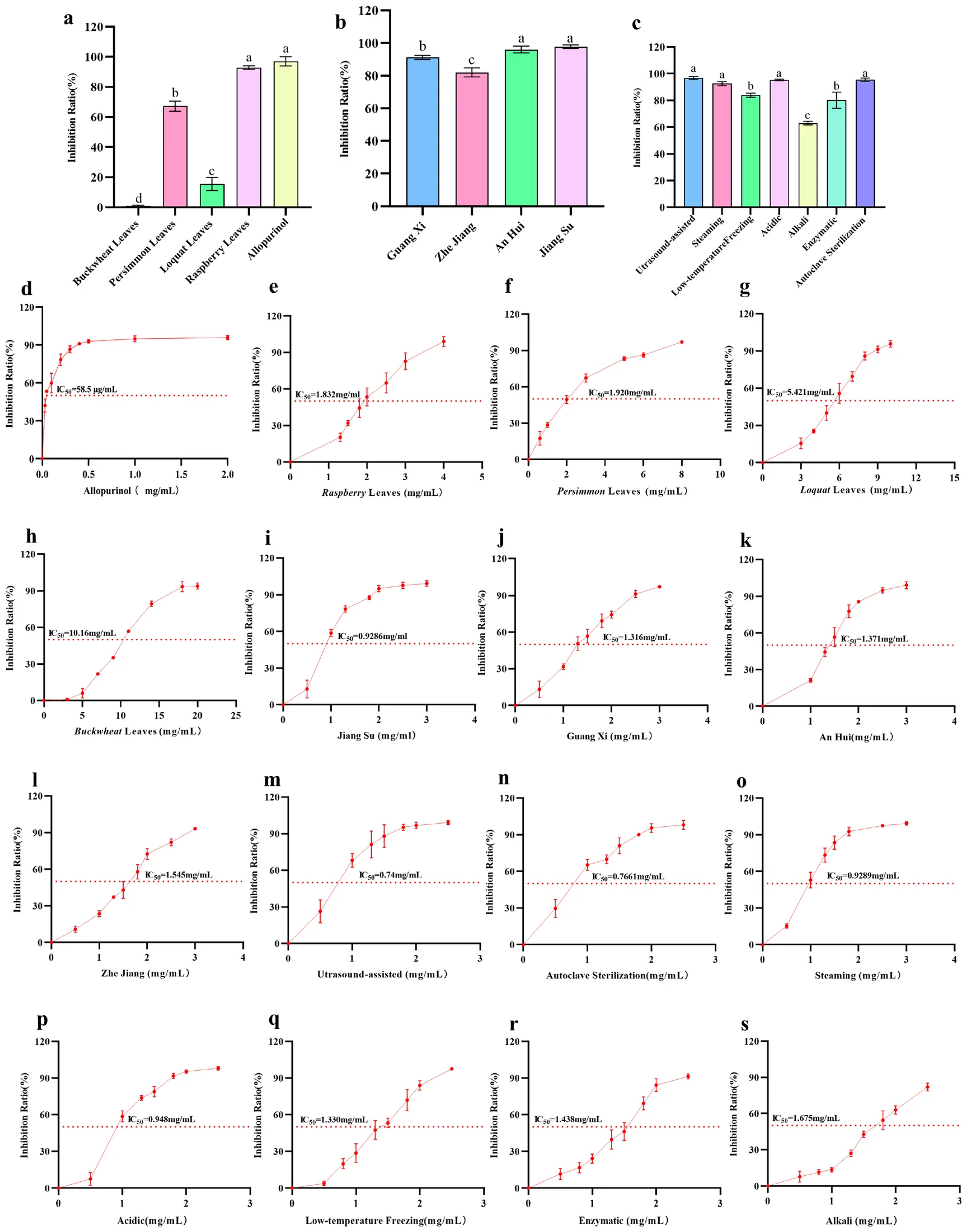
Screening of leaf materials, geographical origins of raspberry leaves, and pre-treatment methods. (**a**) XO inhibitory rates of four leaf species and allopurinol at 3 mg/mL; (**b**) XO inhibitory rates of four geographical origins at 2.5 mg/mL; (**c**) XO inhibitory rates of seven pre-treatment methods at 2 mg/mL; (**d**–**h**) IC_50_ values of four leaf species and allopurinol; (**i**–**l**) IC_50_ values of four geographical origins; (**m**–**s**) IC_50_ values of seven pre-treatment methods. Different lowercase letters indicate significant differences among groups (*p* < 0.05). Data are expressed as mean ± standard deviation (*n* = 3). The dashed line indicates 50% inhibition.

The four leaf extracts exhibited significantly different XO inhibitory activities at 3.0 mg/mL (*p* < 0.05). Raspberry leaf showed an inhibitory rate of 92.89 ± 0.98% (Figure 1a) and an IC_50_ of 1.832 mg/mL, significantly outperforming buckwheat leaf (IC_50_ = 10.16 mg/mL), loquat leaf (IC_50_ = 5.421 mg/mL), and persimmon leaf (IC_50_ = 1.920 mg/mL). The positive control allopurinol exhibited an IC_50_ of 58.5 μg/mL (Figure 1d–h). The terpenoids, coumarins, and amide alkaloids that are abundant in *raspberry* leaves may bind to the XO active center through hydrophobic interactions, thereby exhibiting superior inhibitory activity [14].

In the geographical origin screening, *raspberry* leaves from four origins showed significantly different activities at 2.5 mg/mL (*p* < 0.05) (Figure 1b). Jiangsu origin had the lowest IC_50_ (0.9286 mg/mL), outperforming Guangxi (1.316 mg/mL), Anhui (1.371 mg/mL), and Zhejiang (1.545 mg/mL) (Figure 1i–l); this origin was therefore selected for subsequent experiments.

The seven pre-treatment methods significantly affected the XO inhibitory activity of *raspberry* leaf at 2.0 mg/mL (*p* < 0.05) (Figure 1c). Ultrasound-assisted extraction yielded the lowest IC_50_ (0.74 mg/mL), followed by high-pressure steaming (0.7661 mg/mL), steaming (0.9289 mg/mL), acid treatment (0.948 mg/mL), low-temperature freezing (1.330 mg/mL), enzymatic treatment (1.438 mg/mL), and alkaline treatment (1.675 mg/mL) (Figure 1m–s). The cavitation effect of ultrasound can effectively disrupt cell walls under mild conditions, promoting the release of terpenoids, coumarins, and other active components while avoiding the limitations of thermal degradation, acid–base residue, and the high cost associated with enzymatic hydrolysis [22,29]. Considering activity performance, food safety applicability, and process economy, ultrasound-assisted extraction was selected as the core process for subsequent response surface optimization.

### 3.2. Results of Ultrasound-Assisted Extraction Optimization

#### 3.2.1. Single-Factor Optimization

**Figure 2:**
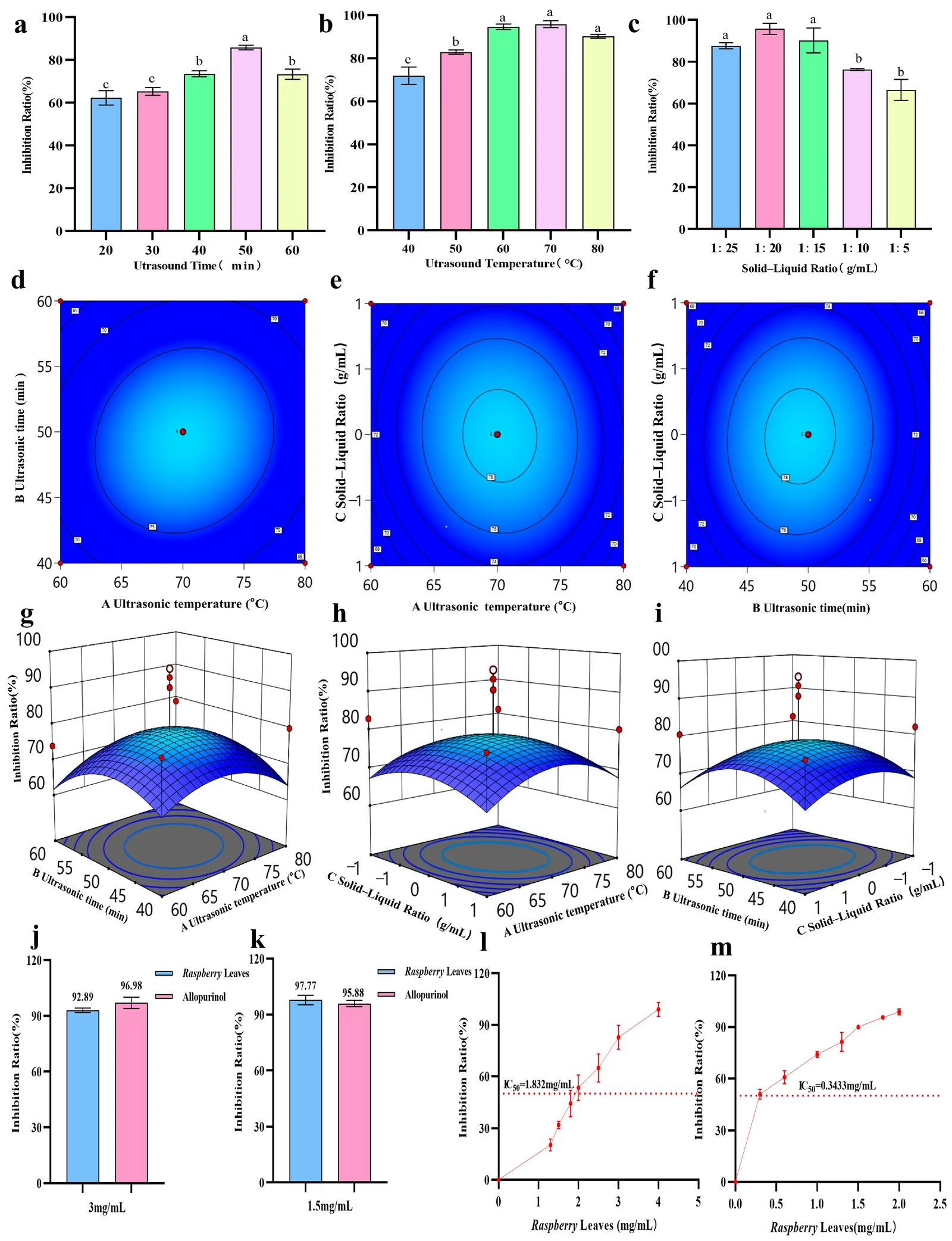
Ultrasound-assisted extraction optimization of raspberry leaves. (**a**–**c**) Single-factor experiments for ultrasound temperature, time, and solid-to-liquid ratio at 1.8 mg/mL, respectively; (**d**–**i**) contour plots and three-dimensional response surface plots from the Box–Behnken design, where the black dot in the center of the contour plots marks the center point (coded level 0) of the design; (**j**–**m**) comparison of XO inhibitory rates and IC50 values before and after optimization. Different lowercase letters indicate significant differences among groups (*p* < 0.05). Data are expressed as mean ± standard deviation (n = 3). The dashed line indicates 50% inhibition.

Single-factor screening tracked XO inhibition at 1.8 mg/mL across temperature, time, and solid-to-liquid ratio (Figure 2a–c). Temperature exhibited a bell-shaped response, peaking at 70 °C (97.47%) before declining sharply at 80 °C. This downturn is consistent with thermal degradation of heat-labile actives—plausibly terpenoids and coumarins subsequently identified as candidate XO-inhibitory compounds (Section 3.5)—as a possible mechanism; however, it must be acknowledged that no direct compositional comparison between extracts prepared at 70 and 80 °C was conducted in this study, and this explanation therefore remains inferential rather than experimentally demonstrated. The decline at 80 °C is unlikely to reflect incomplete extraction, because ultrasound-mediated cavitation remains effective at this temperature and the extraction duration exceeds that required for mass-transfer equilibrium. Extraction time followed a saturating curve, rising steeply to 50 min (86.53%) then plateauing beyond 60 min, suggesting that mass transfer equilibrium was reached and additional sonication risked oxidative degradation without incremental recovery. The RSM ranges were therefore set at 60–80 °C and 40–60 min, respectively.

The solid-to-liquid ratio peaked at 1:20 g/mL (97.50%). Below 1:15 g/mL, insufficient solvent restricted mass transfer and matrix wetting, while above 1:25 g/mL, dilution lowered the effective bioactive concentration in the assay well without improving absolute yield—a dilution artefact particularly relevant to XO inhibition assays where the readout depends on constituent concentration in the reaction mixture rather than total extracted mass. The 1:15–1:25 g/mL range was adopted for RSM optimization.

#### 3.2.2. Response Surface Methodology: Optimization Results

A three-factor, three-level Box–Behnken design was employed to model the combined effects of ultrasound temperature (A), extraction time (B), and solid-to-liquid ratio (C) on XO inhibitory activity. Seventeen experimental runs, including five center-point replicates, were conducted with XO inhibition assayed at 1.5 mg/mL. Inhibitory rates ranged from 80.13% to 97.25% (Supplementary Table S2), with low scatter at the center points, indicating satisfactory experimental reproducibility. Data were fitted to a second-order polynomial equation:

Y = 92.55 − 1.14A + 0.1005B − 0.6796C + 0.2313AB − 2.32AC + 1.61BC − 6.20A^2^ − 6.89B^2^ − 0.9427C^2^

The detailed ANOVA results are presented in Table 1. ANOVA confirmed the model was highly significant (*p* < 0.0001) with a non-significant lack of fit (*p* = 0.4601), supporting adequate fit to the experimental data. The coefficient of determination (R^2^ = 0.9656) and adjusted R^2^ (0.9215) indicated that the model explained over 96% of response variability with minimal inflation from non-informative terms. An inspection of individual terms revealed that none of the linear factors reached conventional significance at *p* < 0.05, although temperature (A) exhibited the strongest marginal effect (*p* = 0.0675), followed by the B × C interaction (*p* = 0.0677). This pattern—non-significant linear terms coupled with highly significant quadratic terms—signals that the response surface is dominated by curvature rather than monotonic slopes, i.e., each factor operates within an optimal window beyond which returns diminish. Notably, the A × C interaction was significant (*p* = 0.0172), indicating that the efficacy of temperature depends on the amount of solvent present: adequate solvent volume is required to dissipate heat and maintain the concentration gradient that drives mass transfer at elevated temperatures.

Among the quadratic terms, A^2^ and B^2^ were highly significant (*p* < 0.0001), confirming parabolic optima for temperature and time with clear peaks. In contrast, C^2^ was not significant (*p* = 0.2368), suggesting that the solid-to-liquid ratio exerted a comparatively weaker curvature effect within the 1:15–1:25 g/mL range, consistent with the broad plateau observed in single-factor screening around 1:20 g/mL.

The contour plots (Figure 2d–i) visualized these relationships, with the A × C interaction displaying the steepest gradient, underscoring temperature and solvent volume as the most influential coupled variables. Predicted optimal conditions were 70 °C, 50 min, and 1:20 g/mL, which aligned closely with the single-factor optima. Three validation runs under these conditions gave 97.77 ± 0.08% inhibition, within 0.5% of the predicted value and comparable to the 97.47% peak observed at 70 °C in single-factor screening. The narrow gap between R^2^ and adjusted R^2^ (ΔR^2^ = 0.044) further supports model reliability and the absence of over-fitting.

#### 3.2.3. Validation of Optimization Efficacy

The IC_50_ of the raspberry leaf extract decreased from 1.832 mg/mL before optimization (conventional decoction, inhibition rate 92.89 ± 0.98% at 3.0 mg/mL) to 0.3433 mg/mL after UAE optimization, representing an approximately 5.34-fold improvement in inhibitory potency with good dose dependency (Figure 2j–m). Under the optimal UAE conditions, the extract exhibited an inhibitory rate of 97.77 ± 0.08% at 1.5 mg/mL, approaching that of the positive control allopurinol (96.98% at 3 mg/mL). Ultrasound-assisted extraction effectively enriched the XO-inhibitory components from raspberry leaves.

### 3.3. Xanthine Oxidase Inhibition Type and Kinetic Analysis

**Figure 3:**
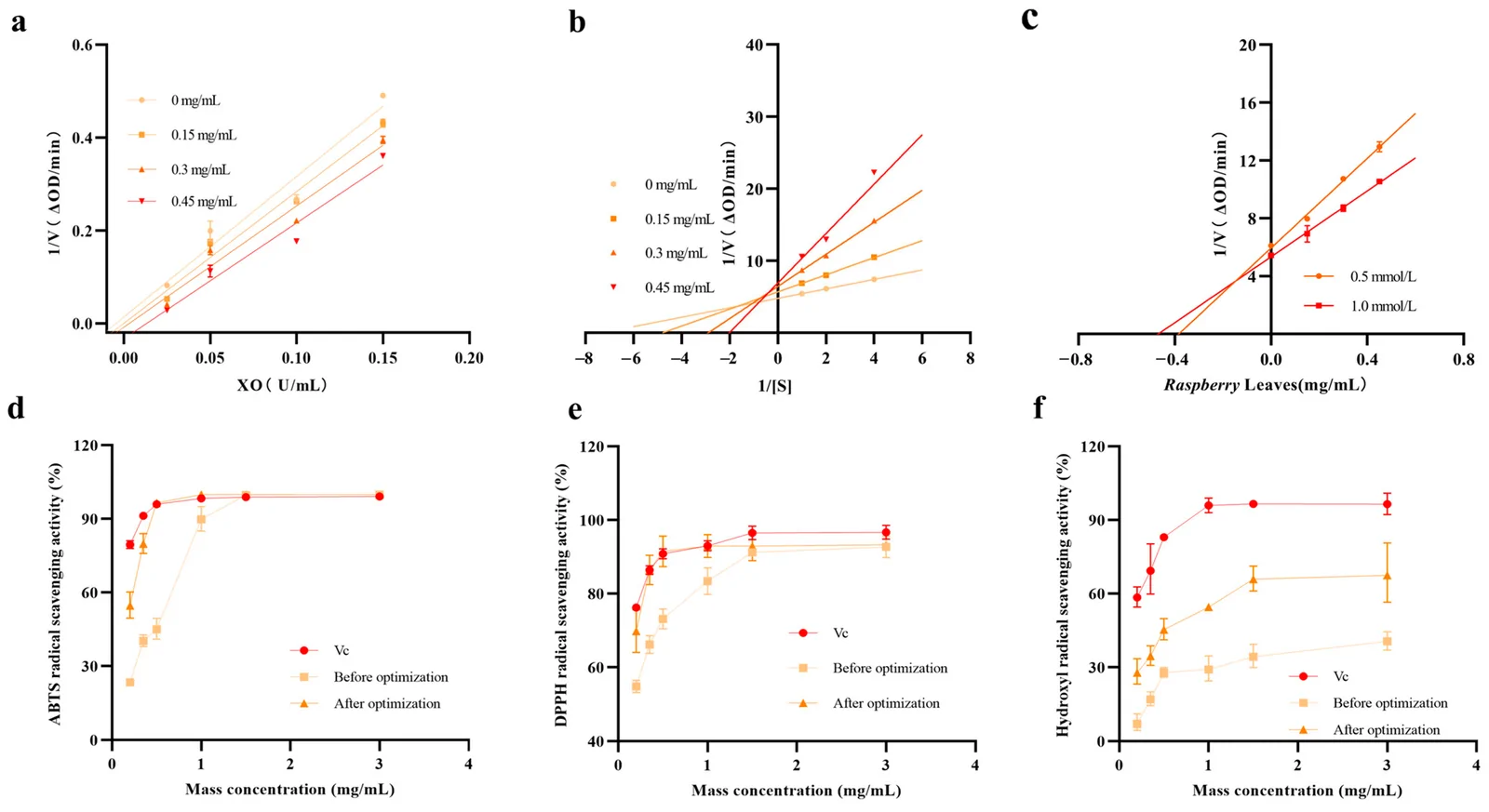
Comparison of bioactivities of raspberry leaf extracts before and after process optimization. (**a**–**c**) XO inhibition effect, inhibition type, and inhibition constant; (**d**–**f**) scavenging capacities against DPPH, ABTS, and hydroxyl radicals. Data are expressed as mean ± standard deviation (*n* = 3).

The reversibility of enzyme inhibition is shown in Figure 3a. At varying extract concentrations, the fitted curves of enzyme concentration versus reaction rate converged toward the origin, with the reaction rate decreasing continuously as sample concentration increased, indicating that the inhibition of XO by raspberry leaf extract was reversible [4]. Lineweaver–Burk double-reciprocal plots showed that the lines for all extract concentrations intersected in the second quadrant, with both slope and y-intercept changing with extract concentration, consistent with reversible mixed-type inhibition kinetics (Figure 3b) [43]. The inhibition constant (*K*_i_) was determined to be 0.147 mg/mL by Dixon plot analysis (Figure 3c). The relatively low *K*_i_ value indicates strong binding affinity between the active components and XO [10]. Collectively, these results demonstrate that raspberry leaf extract reduces XO catalytic activity via a reversible mixed-type mechanism with dose-dependent potency.

### 3.4. Antioxidant Activity Results

Raspberry leaf extract scavenged DPPH, ABTS, and hydroxyl radicals in a dose-dependent manner. UAE optimization lowered the IC_50_ across all three assays: DPPH from 0.1669 to 0.08419 mg/mL (relative antioxidant activity, RAA, increased 1.98-fold from 36.32% to 72.01%); ABTS from 0.4432 to 0.1886 mg/mL (RAA 2.35-fold); and hydroxyl radicals from 4.899 to 0.7598 mg/mL (RAA 6.46-fold) (Figure 3d–f). The parallel improvement in XO inhibition and antioxidant capacity indicates that UAE enriched both bioactive classes simultaneously rather than trading one for the other. This co-enrichment is consistent with the metabolomic profile: terpenoids and coumarins—the candidate XO-inhibitory compounds XO inhibitors identified in the optimized extract—also possess intrinsic radical-scavenging capacity, while the mild extraction temperature (70 °C) preserved heat-labile phenolics and flavonoids that contribute to the antioxidant pool.

The most pronounced gain was observed for hydroxyl radical scavenging (6.46-fold), which carries particular physiological relevance. XO catalysis generates hydrogen peroxide and superoxide, which dismutate to highly reactive hydroxyl radicals (•OH) via Fenton chemistry; these species drive the oxidative tissue damage associated with chronic hyperuricemia [40]. Effective •OH quenching by the optimized extract therefore complements its XO inhibitory activity, addressing both the enzymatic source and the downstream oxidative consequences of purine metabolism dysregulation [39]. Nevertheless, the extract remained less potent than ascorbic acid on a mass basis, reflecting a fundamental kinetic distinction: ascorbate donates hydrogen atoms directly and non-enzymatically, yielding rapid single-electron transfer, whereas plant polyphenols operate through slower, multi-step redox cycling involving metal chelation and regeneration by endogenous antioxidant networks [41]. For functional food applications, this kinetic profile is advantageous: the extract provides sustained, catalytic-style antioxidant coverage compatible with long-term dietary intake, rather than the stoichiometric consumption characteristic of direct antioxidants.

### 3.5. Uhplc-Ms/ms Metabolomics Analysis

**Figure 4:**
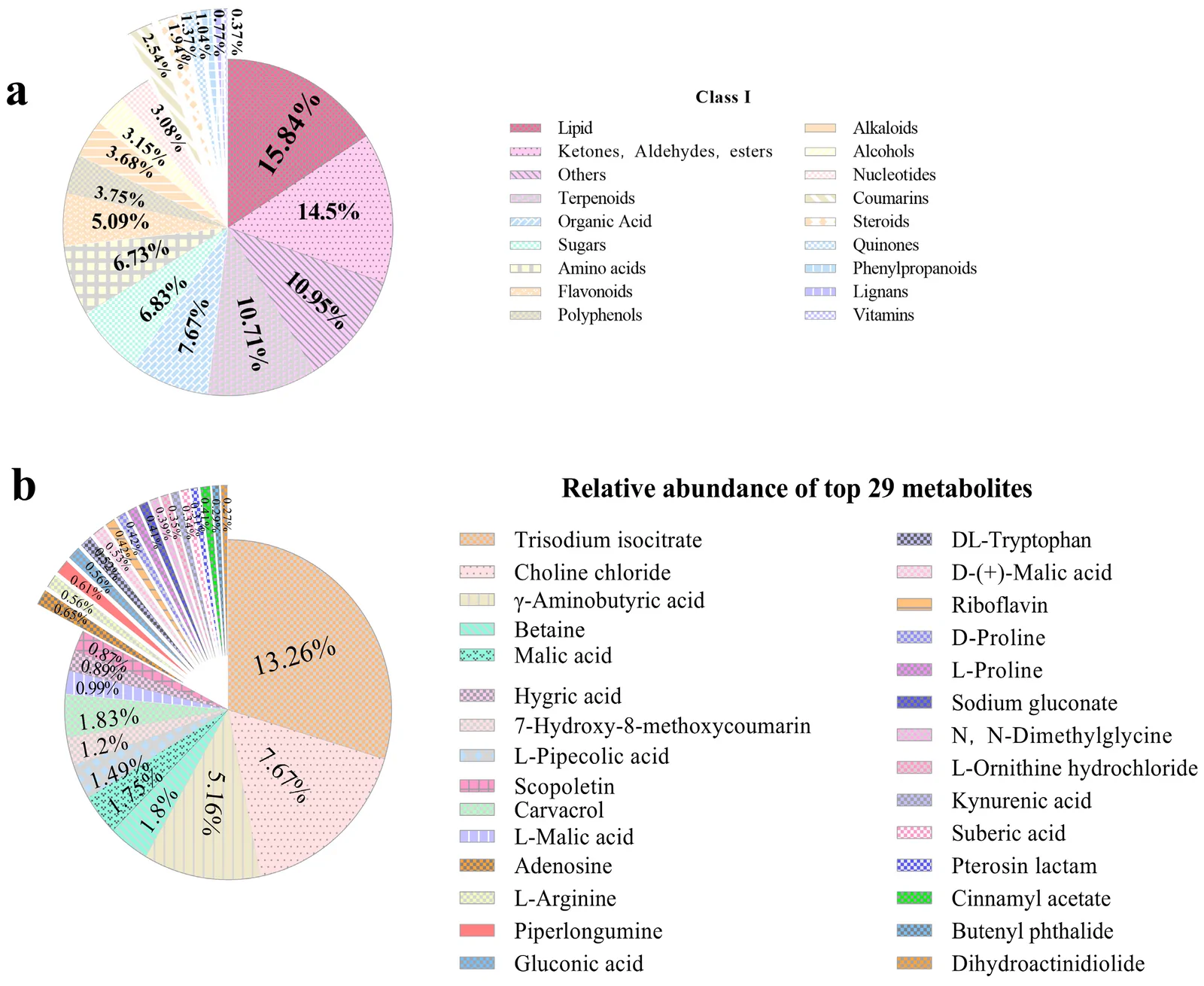
Metabolomics analysis of raspberry leaf extract. (**a**) Classification of identified metabolites; (**b**) top 29 metabolites ranked by relative abundance. Data are expressed as mean ± standard deviation (*n* = 3).

A total of 2987 metabolites were identified by UHPLC-MS/MS widely targeted metabolomics, covering various secondary metabolite classes including lipids, terpenoids, organic acids, amino acids, alkaloids, flavonoids, polyphenols, and coumarins (Figure 4a). Lipids were the most abundant class, followed by ketones/aldehydes/esters and terpenoids; organic acids, alkaloids, flavonoids, and polyphenols collectively constituted the bioactive constituent pool. The top 29 components by relative abundance included organic acids such as isocitric acid, malic acid, and quinic acid; alkaloids such as betaine and stachydrine; amino acids such as DL-tryptophan and L-arginine; and terpenoid and coumarin derivatives including scopoletin, dihydroactinidiolide, carvacrol, and cinnamyl acetate (Figure 4b). These components may directly target XO to inhibit uric acid synthesis and concurrently scavenge free radicals to attenuate oxidative stress, exerting uric acid-lowering and antioxidant effects through multi-component synergy [18,44]. Network pharmacology and molecular docking further indicated that the uric acid-lowering activity of raspberry leaves is not confined to the traditionally recognized flavonoid and polyphenol classes; terpenoids, coumarins, and alkaloids also contribute substantially [44]. Among these, dihydroactinidiolide [45], 7-hydroxy-8-methoxycoumarin [46], and piperlongumine [47] are candidate compounds potentially contributing to XO inhibition. Representative QC-TIC chromatograms and MRM multi-peak chromatograms are provided in Supplementary Materials (Supplementary Figure S1).

### 3.6. Network Pharmacology Analysis

**Figure 5:**
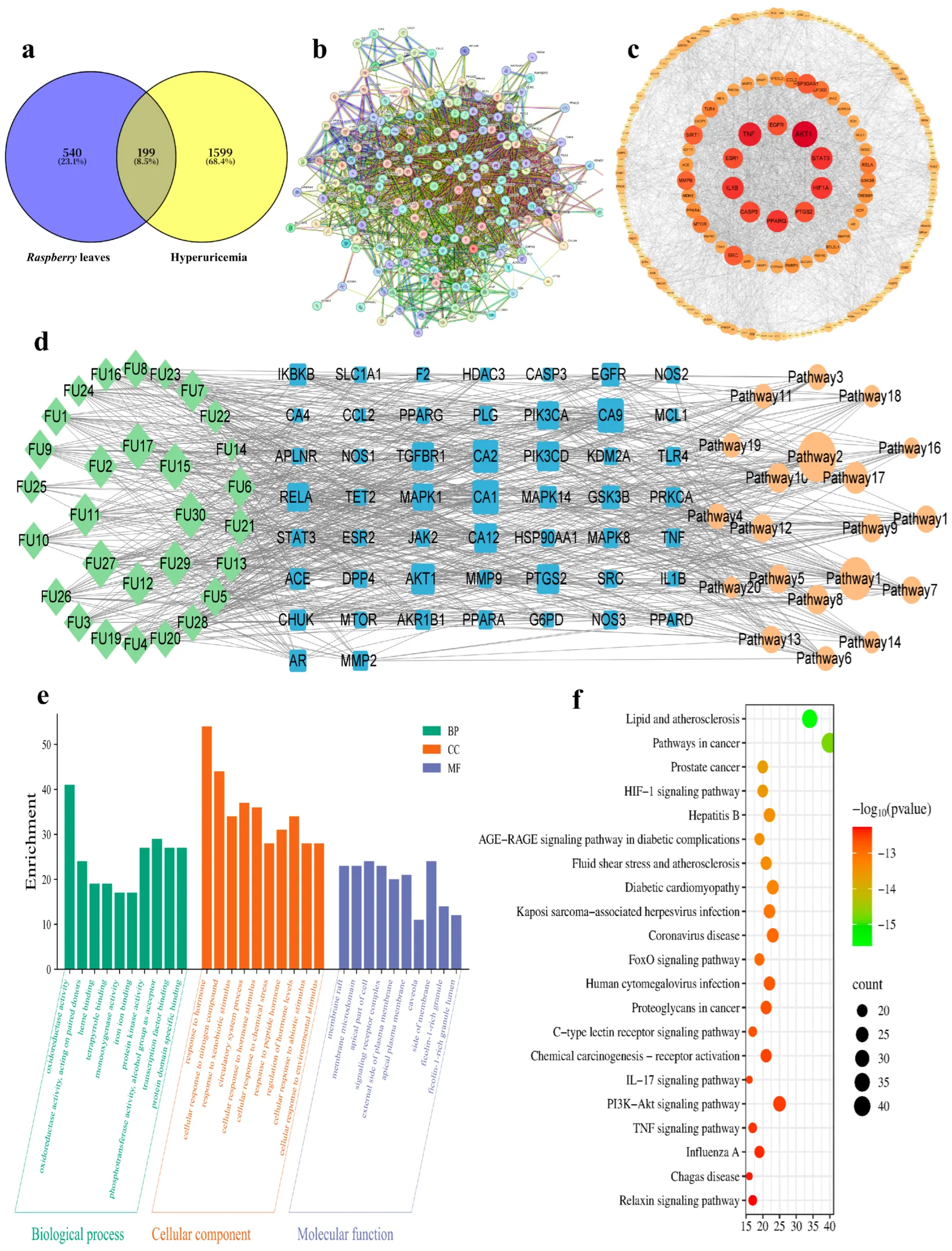
Network pharmacology analysis of raspberry leaf extract against hyperuricemia. (**a**) Venn diagram of intersected targets; (**b**,**c**) PPI network of intersected targets; (**d**) active component–target-pathway interaction network; (**e**) GO functional enrichment; (**f**) KEGG pathway enrichment.

From the 29 metabolites that showed the highest abundance, potential protein targets were predicted. UniProt was used to standardize gene nomenclature and remove duplicates. The resulting target set was then overlapped with hyperuricemia-associated gene lists pulled from GeneCards and OMIM, yielding the candidate targets through which raspberry leaf may act (Figure 5a). These overlapping targets were fed into STRING to build a PPI network scoped to Homo sapiens; singleton nodes were filtered out. Cytoscape handled both visualization and topological scoring. Hub targets were extracted using node degree, betweenness centrality, and complementary topological metrics (Figure 5b,c). The highest-degree hubs included AKT1 (serine/threonine kinase 1), TNF, STAT3 (signal transducer and activator of transcription 3), IL1B (interleukin-1β), and HIF1A (hypoxia-inducible factor 1A). Their involvement pointed to regulatory axes spanning cell proliferation, metabolic homeostasis, inflammation, and oxygen sensing.

GO enrichment was assessed under all three ontologies—biological process, cellular component, and molecular function (Figure 5e). Under biological process, the strongest enrichments were inflammatory-response regulation, oxidative-stress response, and control of cell proliferation plus apoptosis. Cellular-component terms clustered mainly around the plasma membrane, cytoplasm, extracellular region, and mitochondria. Molecular-function hits covered protein binding, kinase activity, cytokine-receptor binding, and transcription-factor binding. This enrichment landscape mirrors the pathophysiology of hyperuricemia, which is marked by activated inflammation, oxidative injury, and metabolic derangement.

KEGG analysis (Figure 5f) flagged six enriched pathways (*p* < 0.05) tied to hyperuricemia: PI3K-Akt, TNF, HIF-1, FoxO, IL-17, and lipid-and-atherosclerosis. PI3K-Akt runs cell metabolism and survival and may improve uric acid handling by adjusting urate-transporter expression [48]. TNF signaling drives inflammation behind kidney damage and gout [49]. HIF-1 turns on when monosodium urate crystals create local hypoxia [50]. IL-17 releases pro-inflammatory factors, worsening joint and kidney inflammation [51]. The lipid pathway tracks the dyslipidemia that often comes with hyperuricemia [52]. Together these pathways form an integrated network through which raspberry leaf could exert multi-target effects on hyperuricemia.

The extract hits several disease nodes at once rather than one isolated target. We mapped metabolites, targets, and pathways onto one network (Figure 5d). Topology showed the classic pattern: one metabolite bound several proteins, and one protein branched into several downstream routes, with overlap among pathways. This layout fits a systems-level mechanism, where raspberry leaf phytochemicals act through multiple overlapping routes rather than a single compound hitting a single target.

### 3.7. Molecular Docking Analysis

**Figure 6:**
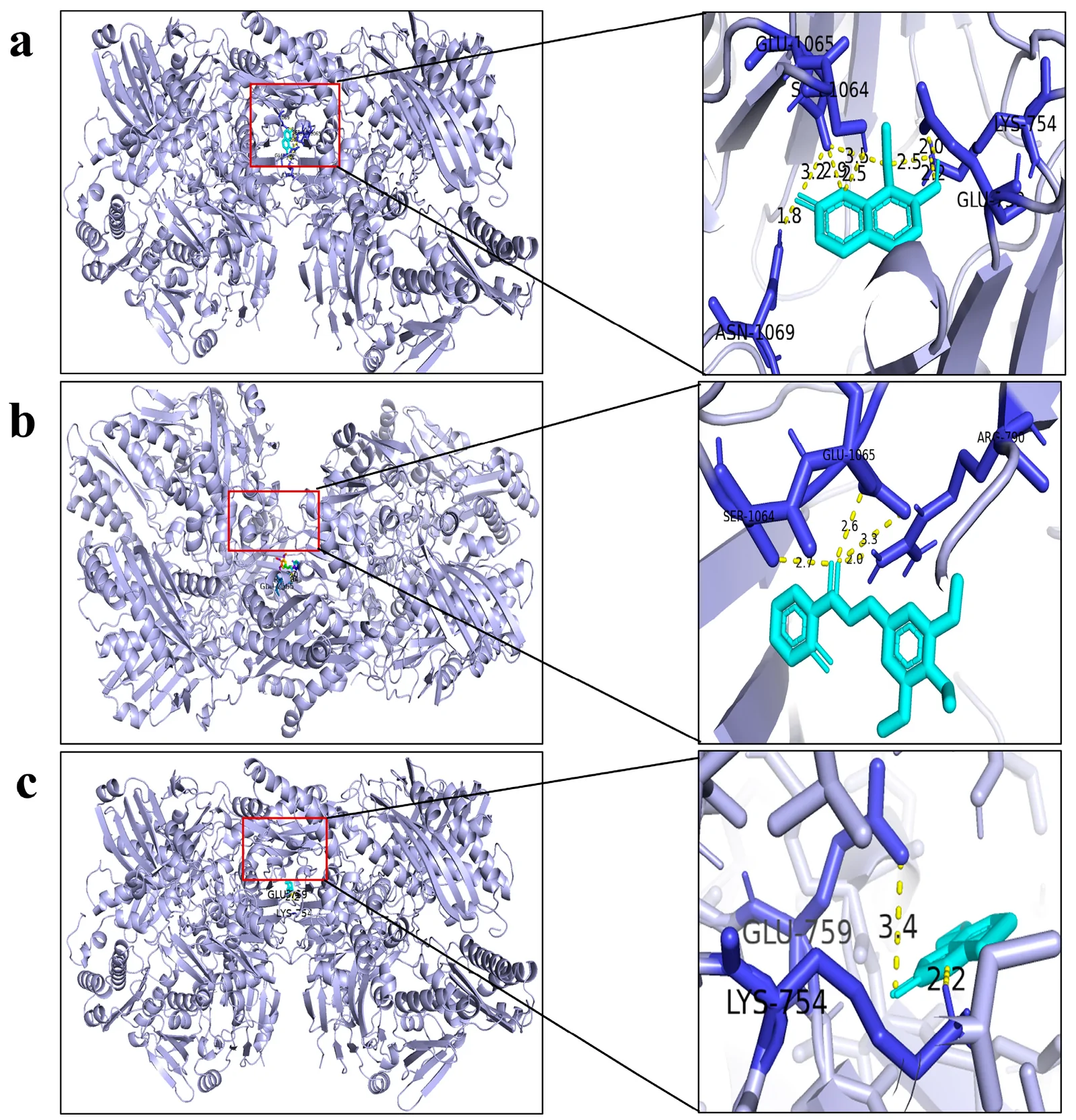
Molecular docking of key metabolites with XO protein (PDB: 3NVY). (**a**) DL-Tryptophan; (**b**) piperlongumine; (**c**) dihydroactinidiolide. The protein is shown in gray/purple cartoon representation, the ligand is displayed in cyan/green sticks, and the binding pocket is highlighted with a red dashed box. Yellow numbers indicate hydrogen bond distances (Å) between the ligand and amino acid residues.

Seven raspberry-leaf metabolites were docked to XO (PDB: 3NVY) (Figure 6a–c). Dihydroactinidiolide, 7-hydroxy-8-methoxycoumarin, and piperlongumine gave binding energies of −6.80, −6.23, and −6.15 kcal/mol; butylidenephthalide, DL-tryptophan, scopoletin, and riboflavin also fell below −5 kcal/mol. All seven met the −5 kcal/mol cut-off for meaningful binding. This finding departs from the standard view that flavonoids dominate plant XO inhibition. In raspberry leaf, terpenoids, coumarins, and alkaloids appear to account for the bulk of the XO blockade. Each ligand occupied the hydrophobic catalytic cavity. DL-tryptophan formed H-bonds with glutamic acid, lysine, and asparagine residues within the XO binding pocket. The docking poses are consistent with the network-pharmacology prediction that multiple compound classes contribute to XO inhibition. They are also compatible with the mixed-type inhibition mechanism established by kinetic analysis (Section 3.3): several compounds bind at or near the active site, which may underlie the competitive component of inhibition, while additional contacts at the periphery of the catalytic cavity may contribute to the non-competitive component. It must be emphasized, however, that molecular docking provides only computational binding poses and cannot by itself establish inhibition kinetics or quantify the relative competitive and non-competitive contributions; these mechanistic features were determined experimentally by Lineweaver–Burk and Dixon plots.

## 4. Conclusions

This study establishes *Rubus chingii* leaf extract as a potent natural source of xanthine oxidase (XO) inhibitors suitable for functional food applications. Among four leafy species evaluated, raspberry leaf exhibited the strongest XO inhibitory activity; Jiangsu-origin material outperformed other geographical sources, and ultrasound-assisted extraction (UAE) surpassed six alternative pre-treatment methods. Response surface optimization identified 70 °C, 50 min, and a solid-to-liquid ratio of 1:20 g/mL as the ideal extraction parameters, yielding an extract with 97.77 ± 0.08% XO inhibition and an IC_50_ of 0.3433 mg/mL—representing a 5.34-fold potency improvement over conventional aqueous decoction. Kinetic analysis confirmed a reversible mixed-type inhibition mechanism (*K*_i_ = 0.147 mg/mL), while parallel antioxidant assessments demonstrated enhanced DPPH, ABTS, and hydroxyl radical scavenging capacity.

Integrating UHPLC-MS/MS widely targeted metabolomics with network pharmacology and molecular docking identified terpenoids, coumarins, and alkaloids as candidate XO-inhibitory compound classes—specifically dihydroactinidiolide, 7-hydroxy-8-methoxycoumarin, and piperlongumine—rather than from the flavonoid and polyphenol fractions conventionally assumed to dominate plant-derived XO inhibition. This finding challenges the prevailing phytochemical paradigm and expands the structural scope of natural XO antagonists beyond phenolic compounds.

The UAE protocol operates under mild conditions, produces no solvent residue, and is readily scalable, positioning the optimized extract as a promising food-grade ingredient for hyperuricemia management. Future work should validate these in vitro findings through animal models and clinical intervention studies to establish dosage–response relationships and long-term safety profiles for human consumption. In addition, isolation and purification of the candidate terpenoids, coumarins, and alkaloids identified herein, followed by in vitro validation of their individual and synergistic XO inhibitory activities using authentic standards, should be prioritized.

## Figures and Tables

**Table 1:** Analysis of variance (ANOVA) for the fitted quadratic model.

Source	Sum of Squares	*df*	Mean Square	*F*-Value	*p*-Value	Significance
Model	439.85	9	48.87	21.86	<0.0001	**
A-Ultrasonic temperature	10.44	1	10.44	4.67	0.0675	
B-Ultrasonic time	0.0808	1	0.0808	0.0362	0.8546	
C-Solid–liquid ratio	3.69	1	3.69	1.65	0.2395	
AB	0.214	1	0.214	0.0957	0.766	
AC	21.54	1	21.54	9.63	0.0172	*
BC	10.42	1	10.42	4.66	0.0677	
A^2^	162.02	1	162.02	72.48	<0.0001	**
B^2^	199.64	1	199.64	89.3	<0.0001	**
C^2^	3.74	1	3.74	1.67	0.2368	
Residual	15.65	7	2.24			
Lack of Fit	6.92	3	2.31	1.06	0.4601	
Pure Error	8.73	4	2.18			
Cor Total	455.5	16				

Note: *R*^2^ = 0.9656, Adjusted *R*^2^ = 0.9215, Coefficient of Variation (C.V.) = 1.74%. ** and * indicate significance at *p* < 0.01 and *p* < 0.05, respectively.

## Data Availability

The original contributions presented in this study are included in the article/Supplementary Material. Further inquiries can be directed to the corresponding authors.

## Supplementary Materials

The following supporting information can be downloaded at: https://www.mdpi.com/article/10.3390/foods15183231/s1, Figure S1: Representative chromatograms obtained from UHPLC-MS/MS analysis; Table S1: Experimental design and factor levels for response surface methodology; Table S2: Experimental design and results of response surface methodology.
