# Supplementary material for: Integrating Metabolomics and Network Pharmacology to Identify Xanthine Oxidase Inhibitors from Raspberry Leaf: Optimization by Ultrasound-Assisted Extraction and Kinetic Characterization
Source: Foods. 2026 Sep 12;15(18):3231. doi: 10.3390/foods15183231 (PMC13606569; doi:10.3390/foods15183231)
Supplement: Supplementary file 1 [file foods-15-03231-s001.zip › foods-4546018-supplementary.pdf]

Table S1

Experimental design and factor levels for response surface methodology.

Coded Levels	A Ultrasonic temperature/ °C	B Ultrasonic time /min	C Solid-liquid ratio/(g/ml)
-1	60	40	1:25
0	70	50	1:20
1	80	60	1:15

Table S2

Experimental design and results of response surface methodology.

Run Order	Factor A: Ultrasonic temperature /°C	Factor B: Ultrasonic time /min	Factor C: Solid-liquid ratio /(g/ml)	Response: Xanthine Oxidase Inhibition Rate (%)
1	-1	-1	0	80.13
2	1	-1	0	80.49
3	-1	1	0	87.17
4	1	1	0	82.85
5	-1	0	-1	88.87
6	1	0	-1	92.51
7	-1	0	1	88.93
8	1	0	1	80.30
9	0	-1	-1	87.16
10	0	1	-1	83.43
11	0	-1	1	82.78
12	0	1	1	85.51
13	0	0	0	95.38
14	0	0	0	97.25
15	0	0	0	92.55
16	0	0	0	93.39
17	0	0	0	94.66

Widely Targeted UHPLC-MS/MS Metabolomics Workflow and Parameters:

1. Sample Preparation

A 50 mg aliquot of the freeze-dried UAE-optimised extract was transferred into a 2 mL centrifuge tube, followed by supplementation with 1,000 μL of methanol-acetonitrile-water solution containing internal standards. The resulting mixture was initially subjected to vortex mixing, subsequently homogenised using a ball mill, and finally ultrasonicated for a short period in an ice-water bath to ensure thorough cell disruption. After centrifugation at 12,000 × g for 15 min at 4 °C, the precipitated insoluble debris was discarded, and the clarified supernatant was filtered through a 0.22 μm PTFE membrane before being loaded onto the LC-MS system. To enable quality-control monitoring, equal volumes of every filtered sample were pooled together to generate a single mixed QC sample.

2. Chromatographic Conditions

Chromatographic separation was carried out using an ACQUITY UPLC HSS T3 column (100 mm

× 2.1 mm, 1.8 μm; Waters, Milford, MA, USA), with column temperature stabilized at 40 °C. The mobile-phase system comprised solvent A (0.1 % formic acid in water supplemented with 5 mM ammonium acetate) and solvent B (acetonitrile containing 0.1 % formic acid). The same mobile-phase mixtures were applied for both positive-ion and negative-ion acquisition modes. The gradient elution profile was configured as follows: starting with 2 % B and sustaining this condition for 1.5 min; linearly raising the proportion of B up to 50 % at 5 min and keeping this state for 4 min; further linear ramp-up to 98 % B at 9 min (1 min hold); switching back to 2 % B within 10.00-11.00 min, followed by re-equilibration until 14 min. The flow velocity was set to 0.35 mL/min, and 4 μL of sample solution was injected for each run.

3. Mass Spectrometry Conditions

Metabolite signals were acquired with a Qtrap 6500+ mass spectrometer (AB Sciex, Framingham, MA, USA) fitted with a Turbo ion-spray ESI source. Data collection was managed via Analyst 1.6.3 software. The ion-source temperature was kept constant at 550 °C; electrospray voltages were adjusted to +5,500 V for positive-ion mode and −4,500 V for negative-ion mode. The values of GS1, GS2 and CUR gas pressures were 50 psi, 55 psi and 35 psi, respectively. Collision-induced dissociation was operated under medium energy level. All measurements were completed under multiple reaction monitoring (MRM) mode. Before sample batch injection, MRM ion transitions were predefined through direct infusion of reference standards whenever accessible. For each ion pair, declustering potential (DP) and collision energy (CE) were individually fine-tuned to achieve optimal signal responses. Throughout data acquisition, target MRM ion pairs were tracked within scheduled time windows matching the elution interval of each individual metabolite.

4. Metabolite Identification

Metabolite identification was performed by matching the acquired MS/MS spectral data against the in-house GB-PLANT metabolite database (MetWare Biotechnology Co., Ltd., Wuhan, China). A metabolite was positively identified when its characteristic precursor ion (Q1), product ion (Q3), and chromatographic retention time (RT) matched the corresponding entries in the database. The identified metabolite list was manually curated to remove redundancies and isotopic adducts.

5. Quantification and Data Normalisation

For quantification, MRM peak areas were extracted using Analyst 1.6.3 software. To correct for inter-sample variation in ionisation efficiency and instrument response, peak areas of the same metabolite across different samples were normalised by internal-standard correction and QC-based inter-sample correction following the procedure described by Fraga et al. (2010). Metabolite abundance is reported as relative quantification values based on corrected peak-area ratios.

6. Software and Instrument Information

Analyst version 1.6.3 was used for instrument control, data acquisition, and peak area extraction. Chromatographic separation was performed on an ACQUITY UPLC system (Waters). MS/MS detection was carried out on a Qtrap 6500+ mass spectrometer (AB Sciex). Metabolite identification was conducted using the in-house GB-PLANT metabolite database provided by MetWare Biotechnology Co., Ltd. (Wuhan, China).

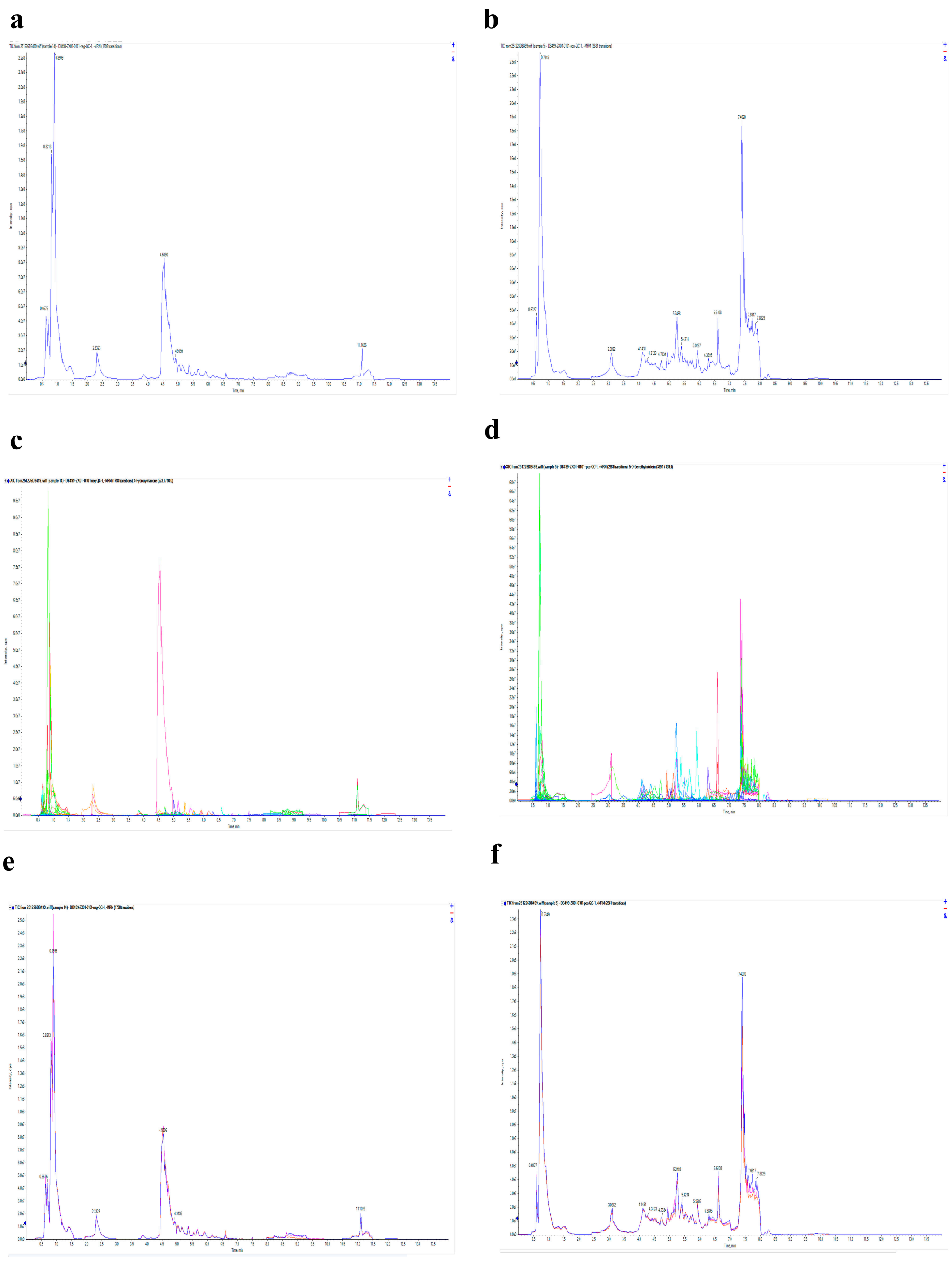

Figure S1. Representative chromatograms obtained from UHPLC-MS/MS analysis. (a, b) total ion chromatograms (TIC) in positive and negative ion modes; (c, d) multiple reaction monitoring (MRM) multi-peak chromatograms in both modes; (e, f) TIC overlays of replicate QC injections demonstrating technical reproducibility.
